# The Microarchitecture of Pancreatic Cancer as Measured by Diffusion-Weighted Magnetic Resonance Imaging Is Altered by T Cells with a Tumor Promoting Th17 Phenotype

**DOI:** 10.3390/ijms21010346

**Published:** 2020-01-05

**Authors:** Philipp Mayer, Alica Linnebacher, Hannah Glennemeier-Marke, Nicole Marnet, Frank Bergmann, Thilo Hackert, Miriam Klauss, Tanja Poth, Matthias M. Gaida

**Affiliations:** 1 Clinic for Diagnostic and Interventional Radiology, University Hospital Heidelberg, 69120 Heidelberg, Germany; Philipp.Mayer@med.uni-heidelberg.de (P.M.); Miriam.Klauss@med.uni-heidelberg.de (M.K.); 2 Institute of Pathology, University Hospital Heidelberg, 69120 Heidelberg, Germany; alica.linnebacher@gmx.de (A.L.); hannah.glennemeier.marke@gmail.com (H.G.-M.); nicole.marnet@unimedizin-mainz.de (N.M.); frank.bergmann@mail.klinikum-darmstadt.de (F.B.); Tanja.Poth@med.uni-heidelberg.de (T.P.); 3 Institute of Pathology, University Medical Center Mainz, JGU-Mainz, 55131 Mainz, Germany; 4 Clinical Pathology, Klinikum Darmstadt GmbH, 64283 Darmstadt, Germany; 5 Department of General, Visceral, and Transplantation Surgery, University Hospital Heidelberg, 69120 Heidelberg, Germany; Thilo.Hackert@med.uni-heidelberg.de; 6 Research Center for Immunotherapy, University Medical Center Mainz, JGU-Mainz, 55131 Mainz, Germany

**Keywords:** pancreatic cancer, tumor microenvironment, IL21, IL26, DW-MRI, ADC

## Abstract

Diffusion-weighted magnetic resonance imaging (DW-MRI) is a diagnostic tool that is increasingly used for the detection and characterization of focal masses in the abdomen, among these, pancreatic ductal adenocarcinoma (PDAC). DW-MRI reflects the microarchitecture of the tissue, and changes in diffusion, which are reflected by changes in the apparent diffusion coefficient (ADC), are mainly attributed to variations in cellular density, glandular formation, and fibrosis. When analyzing the T cell infiltrates, we found an association of a tumor-promoting subpopulation, characterized by the expression of interleukin (IL) 21 and IL26, with high ADC values. Moreover, the presence of IL21^+^ and IL26^+^ positive T cells was associated with poor prognosis. Pancreatic cancers—but not healthy pancreatic tissue—expressed receptors for IL21 and IL26, a finding that could be confirmed in pancreatic cell lines. The functionality of these receptors was demonstrated in pancreatic tumor cell lines, which showed phosphorylation of ERK1/2 and STAT3 pathways in response to the respective recombinant interleukins. Moreover, in vitro data showed an increased colony formation of tumor cells. In summary, our data showed an association of IL21^+^ and IL26^+^ immune cell infiltration, increased ADC, and aggressive tumor disease, most likely due to the activation of the key cancer signaling pathways ERK1/2 and STAT3 and formation of tumor colonies.

## 1. Introduction

Pancreatic ductal carcinoma (PDAC) is a highly aggressive malignancy with a 5-year survival time of about 9% [1]. Late detection of disease, rapid cell growth, and spread, the prevalent abundance of desmoplastic stroma, chemoresistance, and perineural invasion are thought to be major causes for the poor prognosis. Moreover, the immune response to tumors creates a proinflammatory environment that might paradoxically promote tumor progression rather than eliminate the tumor (reviewed in [2,3]). Apparently, cytokines and other mediators derived from the infiltrating immune cells are responsible for alterations of the tumor cells and the tumor microarchitecture, because in PDAC as well as in numerous other cancers, the tumor cells acquire receptors for cytokines and use them for their own benefit. Enhanced proliferation, prolonged survival, and resistance towards chemotherapeutics have been described [4]. The major cause of chemoresistance in pancreatic cancer is its plasticity: tumor-intrinsic mutations, altered tumor cell metabolism, epigenetic changes in tumor cells, and the microenvironment—stroma desmoplasia, immune cell infiltrates, or hypoxia—which induce a resistant tumor phenotype [5]. Therefore, combination therapies with gemcitabine plus nab-paclitaxel or the FOLFIRINOX protocol (a combination therapy containing folinic acid, fluorouracil, irinotecan, and oxaliplatin) are superior compared to the formerly widely used gemcitabine monotherapy (reviewed in [5]). Currently, personalized oncology heads towards specific, targeted (combinatorial) therapies, and also include immunotherapeutic approaches, therapies interfering with cancer stem cells or with distinct cancer-associated pathways or receptors [6,7]. But not only the tumor cell itself may be a target, rather tumor-promoting T cells which among others produce tumor necrosis factor (TNF)-*α*, interleukin (IL)10, IL17A, IL21, and IL26 [8,9,10,11] as possible targets. Recently, we described the expression of the receptor for IL21 on pancreatic tumors, and most importantly, we found that both the presence of IL21 positive T cells and the expression of the respective receptor were associated with a shorter survival time of the patients [10].

Consistent with these findings, in experimental settings targeting the inflammatory microenvironment in PDAC has shown a beneficial effect [8,12,13]. In the present study, we used diffusion-weighted magnetic resonance imaging (DW-MRI) to assess the tumor microarchitecture in relation to the intratumor T cell infiltrate. DW-MRI measures the diffusion of water molecules within tumor tissue and is used in clinical diagnosis for the detection and characterization of pancreatic masses. In recent years, DW-MRI has evolved into a versatile tool for in vivo analysis of tissue microarchitecture, providing additional information compared to histopathology alone [14]. DW-MRI allows for the in vivo analysis of the whole tumor, as opposed to conventional histopathology techniques, which rely on ultra-thin two-dimensional tissue slices, and therefore, do not account for the true 3D structure of the intact (unsliced) tissue and might give not the complete representation of the whole lesion.

Diffusion, as expressed by lower values of the apparent diffusion coefficient (ADC), is usually more restricted in PDAC compared to healthy pancreatic tissue [15]. The variations of ADC-values can partly be attributed to fibrosis [16,17], cellular density [18], or necrosis within the tumors [19]. Moreover, also infiltration of neutrophils expressing Azurocidin was associated with altered water diffusion, caused by reprogramming the fibrotic process of pancreatic stellate cells [20]. A possible connection between tumor-associated T cells, respectively, their cytokines and changes in the tumor microarchitecture, have not been addressed so far. This study is now focused on tumor-infiltrating T cells, particularly those expressing IL21 and IL26, as these are major cytokines produced by tumor-promoting Th-17-like cells. The aim of the current study is to investigate the role of IL21 and IL26 on tumor progression as well as their association with changes in the tumor microarchitecture of PDAC that can be detected in vivo by DW-MRI.

## 2. Results

### 2.1. DW-MRI in Patients with PDAC

**Figure 1:**
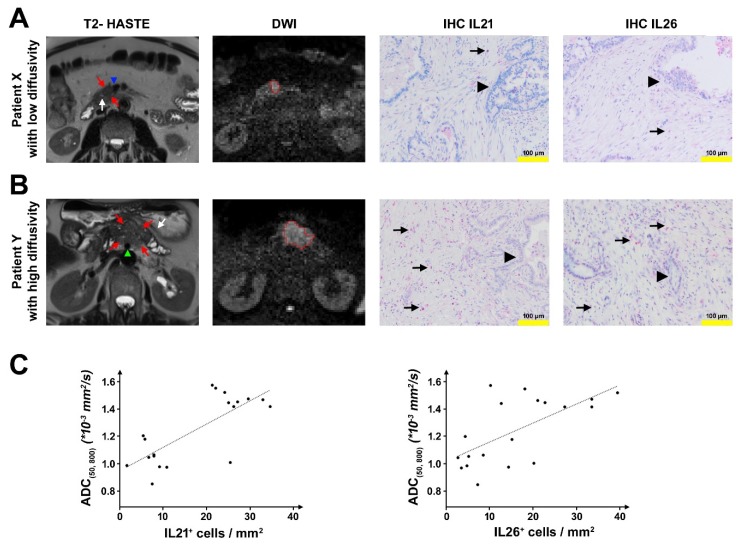
Radiological–pathological correlation. (**A**) Representative patient with a low apparent diffusion coefficient (ADC)_(50, 800)_. Axial T2 half-Fourier acquisition single-shot turbo spin echo (HASTE) image shows a mildly hypointense lesion in the medial part of the uncinate process/ pancreatic head (red arrows) with direct contact to the superior mesenteric vein (blue arrowhead) and in proximity to the main pancreatic duct which is not dilated (white arrow). Diffusion-weighted image (DWI, b = 800 s/mm^2^) with the freehand volume of interest (VOI) from Reader 1 (red) surrounding the hyperintense lesion. Mean ADC_(50, 800)_ for both Readers was 1.0469 × 10^−3^ mm^2^/s. Immunohistochemistry (IHC) shows low numbers of IL21^+^/IL26^+^ cells/mm^2^ (6.67 IL21^+^ cells/mm^2^ and 2.5 IL26^+^ cells/mm^2^) (arrow: red-stained IL21 or IL26 positive cells; arrowhead: tumor cells). (**B**) Representative patient with high ADC_(50, 800)_. Axial T2 HASTE image shows a mildly hyperintense lesion in the pancreatic body (red arrows) with direct contact to the superior mesenteric artery (green arrowhead), upstream dilatation of the main pancreatic duct and concomitant parenchymal atrophy (white arrow). Diffusion-weighted image (DWI, b = 800 s/mm^2^) with the freehand VOI from Reader 1 (red) surrounding the hyperintense lesion. Mean ADC_(50, 800)_ for both Readers was 1.4172 × 10^−3^ mm^2^/s. IHC shows high numbers of IL21^+^/IL26^+^ cells/mm^2^ (26.25 IL21^+^ cells/mm^2^ and 27.08 IL26^+^ cells/mm^2^) (arrow: red-stained IL21 or IL26 positive cells; arrow head: tumor cells). (**C**) Left: Dependency of the ADC_(50, 800)_ on the number of IL21^+^ cells/mm^2^. Linear regression model: $ADC_{\left(50,\;800\right)}\left[\frac{mm^{2}}{s}\right]=0.0000171\left[\frac{mm^{4}}{s}\right]*IL26^{+}cells\left[\frac{1}{mm^{2}}\right]+0.000947\left[\frac{mm^{2}}{s}\right]$, Goodness of fit: $R^{2}=0.5770$; Right: Dependency of the ADC_(50, 800)_ on the number of IL26^+^ cells/mm^2^. Linear regression model: $ADC_{\left(50,\;800\right)}\left[\frac{mm^{2}}{s}\right]=0.0000140\left[\frac{mm^{4}}{s}\right]*IL26^{+}cells\left[\frac{1}{mm^{2}}\right]+0.0010235\left[\frac{mm^{2}}{s}\right]$, Goodness of fit: $R^{2}=0.4196$.

MRI scans with diffusion-weighted imaging (DWI), which were acquired on the day before surgery, were analyzed by two experienced abdominal radiologists in 19 patients (MRI cohort). The intraclass correlation coefficient (ICC) indicated a good concordance between the ADC_(50, 800)_-measurements by the two readers (ICC = 0.8886) and was similar to the ICCs for ADC-measurements in PDAC reported by Mayer et al. [20] and Wiggermann et al. [21]. Mean ADC_(50, 800)_ values for both readers ranged from 0.8499 × 10^−3^ mm^2^/s to 1.5723 × 10^−3^ mm^2^/s (mean: 1.2446 × 10^−3^ mm^2^/s) which are within the same range as reported in previous studies [15,22]. Tissue specimens of the patients from this MRI cohort were analyzed for infiltrated T-cells expressing IL21/IL26. The number of T cells varied among the patients. Compared to patients with low ADC_(50, 800)_ (defined as < 1.3 × 10^−3^ mm^2^/s), patients with high ADC_(50, 800)_ (defined as ≥ 1.3 × 10^−3^ mm^2^/s) had significantly higher numbers of IL21^+^ cells/mm^2^ (median: 7.71 versus 26.25 IL21^+^ cells/mm^2^, *p* < 0.001) and IL26^+^ cells/mm^2^ (median: 6.04 versus 22.50 IL26^+^ cells/mm^2^, *p* = 0.002) (Figure 1). The content of tumor cells and expanse of desmoplastic stroma was determined within the two groups, revealing no differences.

Conversely, ADC_(50,800)_ values were higher in patients with high numbers of IL21^+^/IL26^+^ cells (>10 cells/mm^2^) compared to patients with low numbers of IL21^+^/IL26^+^ cells (median ADC_(50, 800)_: 1.4496 × 1 ^−3^ mm^2^/s vs. 1.0513 × 10^−3^ mm^2^/s for IL21, *p* = 0.008; median ADC_(50, 800)_: 1.4430 × 10^−3^ mm^2^/s vs. 1.0513 × 10^−3^ mm^2^/s for IL26, *p* = 0.058).

### 2.2. Analysis of Tumor-Infiltrating T Cells in PDAC

**Figure 2:**
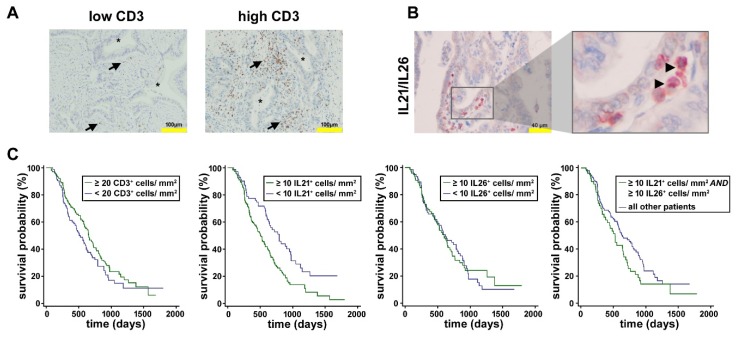
Tumor-infiltrating T cells in pancreatic ductal adenocarcinoma (PDAC). (**A**) Depicted are example pictures from patients with low and high numbers of CD3^+^ cells/mm^2^. Brown cells with an arrow: CD3^+^; asterisk: tumor cells. (**B**) Double staining for IL21 (brown) and IL26 (red) shows co-localization (arrow: double positive brown/red cells). (**C**) Kaplan-Meier curves. The mean survival of patients with a high number of CD3^+^ cells was non-significantly longer than of patients with a low number of CD3^+^ cells. PDAC patients with high IL21 infiltrate had significantly shorter survival than patients with low IL21 infiltrate. No convincing difference for the survival of patients with low versus high IL26 infiltrate was seen, whereas patients with high numbers of IL21^+^ AND IL26^+^ cells survived shorter compared to all other patients.

In the tissue of PDAC patients (*n* = 199), infiltrating T cells, identified by the expression of CD3, were found, though to a varying degree. Patients with high numbers of CD3^+^ cells (≥20/mm^2^) survived longer compared to patients with low numbers of CD3^+^ cells (<20/mm^2^; median 653 vs. 525 days, *p* = 0.144) (Figure 2). In the same tissue, T cells expressing IL21 and IL26, representing signature cytokines of so-called Th17-like cells, were counted. Double staining revealed that in the majority of cells, IL21 and IL26 were co-expressed, and accordingly, there was a close but not absolute correlation between IL21 and IL26 expression (Figure 2). There was a weak but significant positive rank correlation between the numbers of IL21^+^ and IL26^+^ cells/mm^2^ (*r_s_* = 0.227, *p* = 0.020).

A dense IL21 infiltrate (≥10 IL21^+^ cells/mm^2^) was associated with shorter survival time (median 509 days) when compared to patients with low IL21 infiltrate (<10 IL21^+^ cells/mm^2^, median 791 days, *p* < 0.009), in line with our findings reported previously [10]; for IL26 positive T cells, no marked difference was seen (median 604 vs. 626 days; *p* = 0.942). Patients with high numbers of IL21^+^ and IL26^+^ cells (≥10 cells/mm^2^, each) survived shorter than all other patients (median 528 vs. 642 days; *p* = 0.074). At first glance, these data are in apparent contradiction to the observation that CD3^+^ T cells are associated with better survival. However, there was no significant rank-correlation between CD3^+^ T cells and IL21^+^ cells (*r_s_* = 0.013, *p* = 0.856), and only a weak correlation between CD3^+^ and IL26^+^ cells (*r_s_* = 0.246, *p* = 0.001), in line with the fact that although all T cell are CD3 positive, only a distinct population of these cells produce the tumor-promoting cytokines IL21 and IL26. 

Of note, the numbers of IL21 or IL26 positive T cells were not significantly correlated to tumor size, histological grading, or lymph node positivity (*p* ≥ 0.2510).

### 2.3. Expression of Receptors for IL21 and IL26 in PDAC

**Figure 3:**
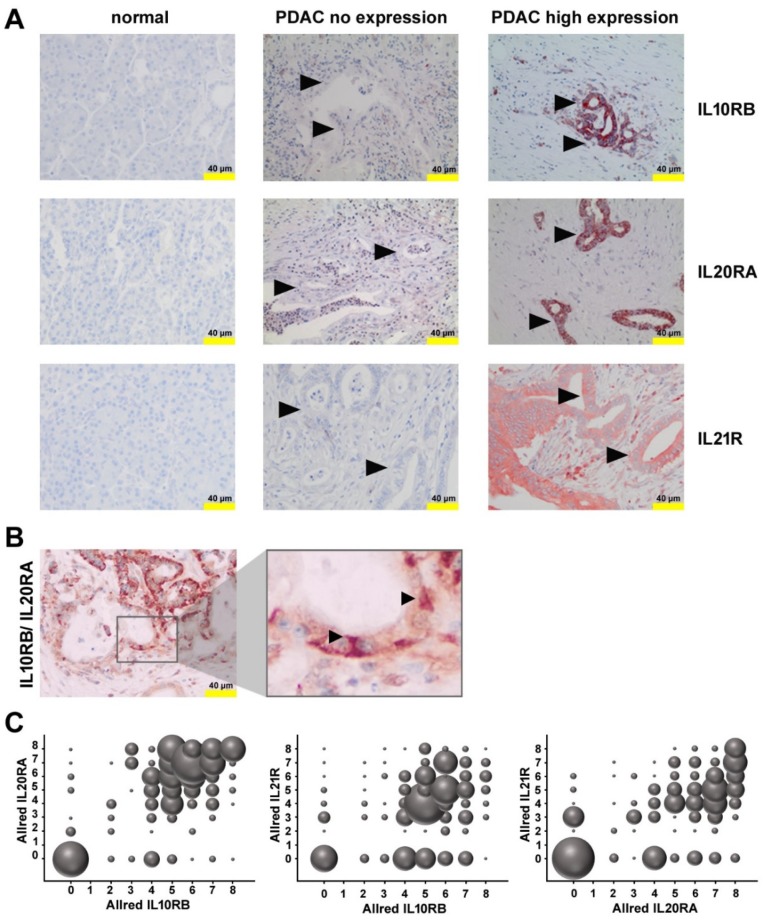
Receptors for IL21 and IL26 in PDAC. (**A**) Immunohistochemistry (IHC) of PDAC samples with no expression and high expression of IL10RB, IL20RA, or IL21R, in comparison to non-neoplastic pancreatic parenchyma. Arrow head: tumor cells (**B**) Double staining for IL10RB (red) and IL20RA (brown) shows co-localization (arrow, red-brown cells). (**C**) Bubble charts show the dependencies of the Allred score for IL20RA from Allred IL10RB (left), Allred IL21R from Allred IL10RB (middle), and Allred IL21R from Allred IL20RA (right). The diameter of the bubbles indicates the frequency of occurrence.

Expression of the receptor for IL21 by pancreatic tumor cells was shown previously [10] by our group and could be confirmed in this study for the here used cohort of patients (Figure 3). There was a trend towards shorter overall survival (OS) time in patients with high IL21R expression (score 5 to 8, median 583 days) compared to patients with low IL21R expression (scores 0 to 4, median 608 days), although the difference was not statistically significant according to the log–rank test (*p* = 0.300). 

IL26 binds to a heterodimeric receptor, consisting of IL10RB and IL20RA, which is highly specific for IL26, whereas the individual subunits of the receptor are also components of other cytokine receptors (e.g., IL10 or IL19). Both IL10RB and IL20RA were found on tumor cells (Figure 3). There was a weak—though statistically significant—correlation between IL10RB and IL20RA expression when the Allred immunoreactivity score was analyzed (*r_s_* = 0.382; *p* < 0.001; regarding the distribution *r_s_* = 0.358; *p* < 0.001, and regarding the intensity *r_s_* = 0.408; *p* < 0.001), compatible with a co-expression of these receptors, but not ruling out expression of the subunits in other combinations. Co-expression of these receptors could be demonstrated using two-color immunohistochemistry.

Patients with high immunoreactivity scores for both, IL10RB und IL20RA, (score ≥ 5) had a shorter survival time (median 577 vs. 632 days; *p* = 0.067). Of note, when calculated separately for each receptor, patients with a high immunoreactivity score for IL10RB (5 to 8) survived longer compared to patients low immunoreactivity IL10RB score (0 to 4) (median 626 vs. 588 days; *p* = 0.491), but conversely, high immunoreactivity score for IL20RA (5 to 8) was associated with shorter survival time (median 583 vs. 613 days; *p* = 0.146), although IL10RB and high IL20RA expression was not associated with tumor size, grading, or lymph node status. 

Inflammation determined by the number/mm^2^ of CD68^+^ cells (monocytes/macrophages) or Azurocidin^+^ cells (polymorphonuclear neutrophils) was less in patients with high score for IL10R AND IL20RA (median/ mean 29.1/ 33.1 CD68^+^ cells/mm^2^ *versus* 38.2/ 40.2 CD68^+^ cells/mm^2^, *p* = 0.029; median/mean Azurocidin^+^ cells/mm^2^ versus 5.4/12.5 versus 6.0/16.7 Azurocidin^+^ cells/mm^2^, *p* = 0.350, Supplementary Figure S1).

### 2.4. Expression of Functional IL10RB and IL20RA on Pancreatic Tumor Cells Lines

**Figure 4:**
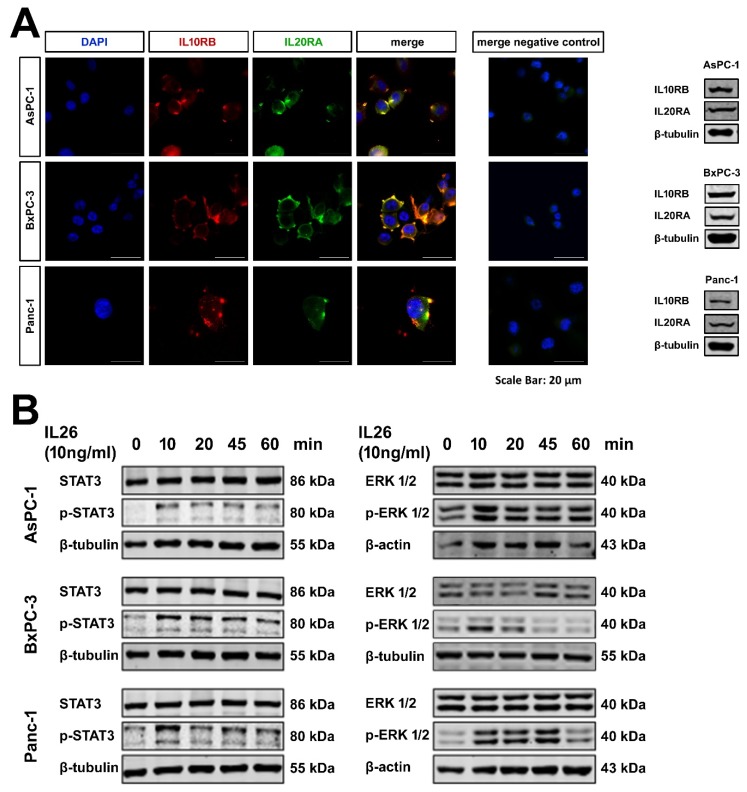
PDAC cell lines. (**A**) Laser scan microscopy and Western blotting of the PDAC cell lines AsPC-1, BxPC-3, and Panc-1 incubated with anti-IL10RB and anti-IL20RA antibodies shows expression and colocalization of both receptors in all three cell lines. (**B**) Western blotting of PDAC cells shows IL26 induced phosphorylation of STAT3 (left panel) and ERK1/2 (right panel) in a time-dependent manner.

IL21R is expressed on pancreatic tumor cells and activates key signaling pathways, as shown previously [10]. To verify the expression of IL10RB and IL20RA on tumor cells, three cell lines, AsPC-1, BxPC-3, and Panc-1 were studied. By Western blotting, IL10RB and IL20RA could be detected in all three cell lines. Laser scan microscopy showed a co-localization of IL10RB and IL20RA (Figure 4). Recombinant IL26 induced phosphorylation of STAT3 and ERK1/2 in a time-dependent manner, indicating that the receptor was functional (Figure 4). The p38 pathway was not activated.

### 2.5. Effect of IL21 and IL26 on Colony Formation

**Figure 5:**
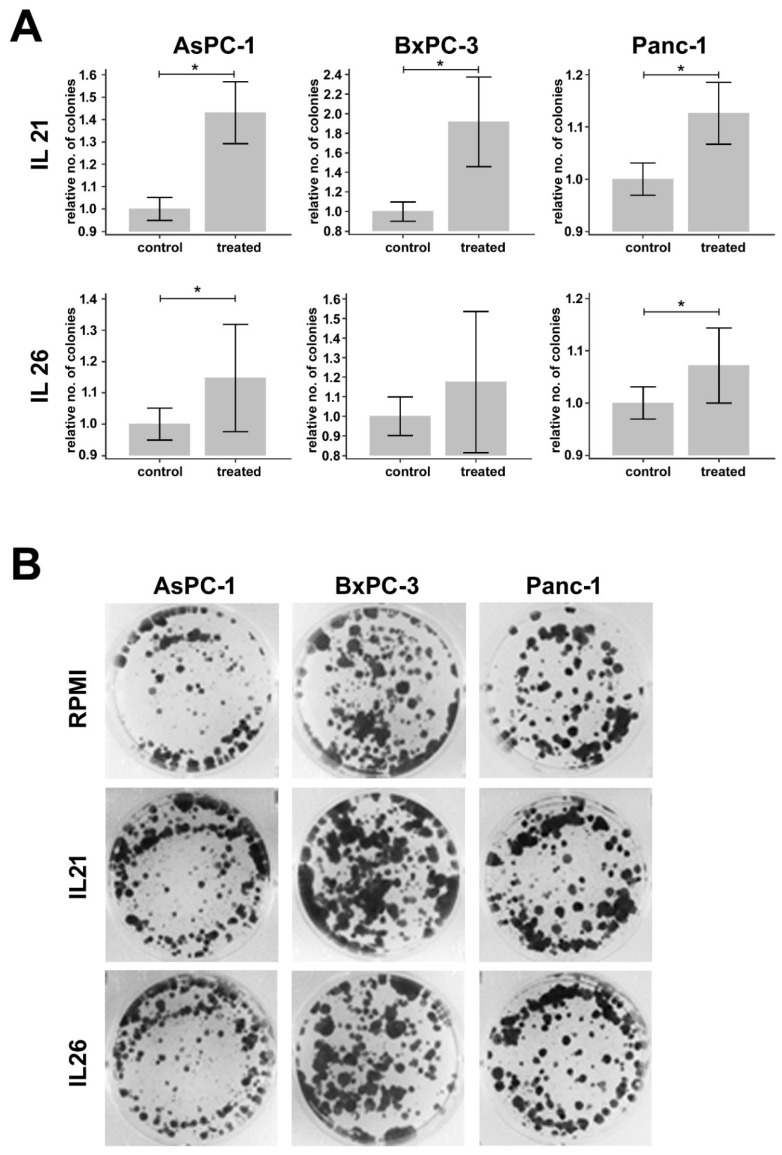
Results of the colony formation assays. (**A**) Bar graphs show relative differences in the number of colonies in PDAC cell lines (AsPC-1, BxPC-3, and Panc-1) treated with IL21 (upper row) or IL26 (lower row) compared to controls. The number of colonies is normalized to the mean number of colonies of controls from the respective day of the experiment. Differences that are statistically different (*p* < 0.05), according to the Mann–Whitney U test, are marked with *. (**B**) Depicted are representative pictures of wells with AsPC-1, BxPC-3, and Panc-1 cells treated with IL21 or IL26 versus representative controls.

To answer the question of whether stimulation of tumor cells by IL26 alters cell function, colony formation was tested in response to IL26. For comparison, IL21 as “relative cytokine” secreted by Th17-like cells was used in the present study because we had shown previously that it enhances tumor cell invasion [10]. Under similar experimental conditions, again, an effect for IL21 was seen, but only a weaker response to IL26 (data summarized in Figure 5).

## 3. Discussion

Diffusion-weighted magnetic resonance imaging (DW-MRI) is a diagnostic tool widely used in pancreatic cancer to assess the whole pancreatic tumor mass, histologically consisting of the tumor cells itself and of a prominent fibro-inflammatory stroma [19]. DW-MRI basically measures the water diffusion within the tissue and is quantitated by the so-called “apparent diffusion coefficient” (ADC). Diffusion is a random process related to random thermal motion (Brownian motion). Within tissue, diffusion can be reduced by structural barriers, such as stromal collagen and cell membranes. Hence, DW-MRI reflects the microstructure of tissues. Tumors usually have a lower ADC compared to healthy tissue since increased cellular density and fibrosis result in a more restricted water diffusion [14]. Different other histological parameters of PDAC have been associated with changes in the ADC, such as necrosis, which may be a component of larger tumors or a result of radiochemotherapy [19]. The relationship between parameters from histopathology and DW-MRI is not always uniform. For instance, an initial study suggested that collagenous fibers are primarily responsible for diffusion abnormalities in PDAC with a negative correlation of the proportion of collagenous fibers and the ADC [17]. However, a later study revealed that the interrelation between fibrosis and DW-MRI in PDAC is more complex than initially expected. Although fibrosis is partly responsible for diffusion restriction in PDAC, diffusion is not intuitively related to the degree of fibrosis, but rather to the combination of glandular structures and surrounding fibrosis [16].

A recent study further illuminated the relationship between the complex microarchitecture of PDAC and DW-MRI and found that infiltrates of tumor-promoting neutrophils are associated with alterations of growth patterns and subsequently of water diffusion [20]. In addition to neutrophils, the immune microenvironment of PDAC also shows increased infiltrates of T cells. In the current study, we were looking for a link between infiltrated T cells and tumor microarchitecture analyzed using DW-MRI and quantitated by the ADC. We focused on the so-called Th17-like cell subpopulation, which was described as a tumor-promoting infiltrated cell type in PDAC [8,9,23]. These cells produce various tumor-promoting cytokines, including TNF-α, IL10, and IL17A, and also the less well-studied cytokines such as IL21 and IL26. Both IL21 and IL26 are found in PDAC tissue, and we demonstrated that IL21 and IL26 infiltrates were significantly lower in patients with restricted diffusion (as expressed by low ADC-values) than in patients with high diffusivity (as expressed by high ADC-values). Conversely, tumors with high diffusivity in DW-MRI, which indicates low compactness of the tissue [14], had more IL21 and IL26 positive cells than patients with low diffusivity. Apparently, Th17-like cells alter the microarchitecture of the tumor towards lower tissue density and thus the water diffusivity in tissues. Hence, in a larger cohort of around 200 patients, we assessed IL21 and IL26 expression and found co-expression within the cells. Though statistically significant, the correlation was not absolute. The images confirm that in addition to double-positive cells, there are T cells expressing only one of these cytokines. Possibly, the kinetics of synthesis or release varies between the cytokines, and possibly also their stability in human tissue.

The discrepancy of IL21 versus IL26 expression could also explain why there was a definite association between IL21 expression and a shorter patients’ survival time, but no significant association of IL26 with overall survival. The fact, however, remained that the IL21/IL26 co-expressing T cell subpopulation was associated with poor survival, supporting the tumor-promoting activity of the particular Th17-like phenotype and their cytokines, quite in contrast to the observation that high numbers of CD3^+^ T cells were rather beneficial. These data go in line with a previous study, where dense numerical infiltrates of CD3^+^ T cells were associated with prolonged survival. The cohort also shows that, if the number of these T cells were activated via an alternative p38 pathway, and T cells were consequently skewed towards the production of IL17-signature cytokines, such as IL17A or IL21, a particularly aggressive disease was seen [8]. These data further support the notion that the determination of T cell subtypes and their corresponding cytokine profile give important prognostic information.

To explain possible action of IL21 and IL26, we analyzed PDAC tissue for expression of the respective receptors and confirmed the expression of the receptor for IL21, as we reported previously [10]. As a novel finding, we now report the expression of receptors for IL26 on human PDAC. IL26 signals via a heterodimeric receptor consisting of the IL10 receptor 2 (IL10R2, also known as IL10 receptor beta (IL10RB)), and the IL20 receptor 1 (IL20R1, also IL20 receptor alpha (IL20RA)). While IL10RB is widely expressed on hematopoietic cells, IL20RA usually occurs only in activated immune cells; expression of both receptors on tumor cell lines and tissues has been described [24]. In pancreatic tumor tissue as well as in pancreatic tumor cell lines, IL10RB and IL20RA were co-expressed and enable IL26 signaling. The presence of IL10RB/IL20RA in tissue was associated with shorter survival time, compatible with the notion that IL26 signaling per se contributes to tumor progression. Of note, when only IL10RB was considered, the opposite appeared to be true. A possible explanation is that IL10RB does not exclusively associate with IL20RA [25].

Functionally, in pancreatic cell lines, IL26 triggered the Janus kinase-signal transducer and activator of transcription (JAK-STAT) pathway, resulting in rapid STAT3 phosphorylation, consistent with data derived from other cells and cells lines, respectively [25]. Moreover, phosphorylation of the MAP kinase ERK1/2 could be seen. IL26 is mainly known for its proinflammatory and antimicrobial/ antiviral activities. There is, however, evidence that IL26 promotes the proliferation and survival of gastric cancer cells [26]. In our experimental setting, we could demonstrate and increased tumor colony formation upon long term incubation with IL21 and IL26, and thus an increased ability to form more and larger vital tumor cell clusters. This may be an explanation of changes in ADC in patients with high and low densities of IL21 and IL26 positive cells, as well as patients’ survival. Moreover, the present data cannot rule out indirect effects of the two cytokines within the tumor microenvironment, such as stimulation of cytokine secretion by other cells, in vivo. 

In conclusion, we report the infiltration of tumor-promoting Th17-like cells, expressing their related cytokines IL21 and IL26 and their association with particularly aggressive disease. These cytokines interact with pancreatic tumor cells and activate key cellular pathways and thus possibly alter the tumor architecture as determined by changes in tissue water diffusion that are measured by MRI. In that, DW-MRI is a non-invasive method that indicates the infiltration of tumor-promoting T cells. 

## 4. Materials and Methods

### 4.1. DW-MRI

The radiological information system (RIS) of the Clinic for Diagnostic and Interventional Radiology of the University Hospital of Heidelberg was searched retrospectively for PDAC patients who had undergone a DW-MRI scan with *b*-values 50 and 800 s/mm^2^ of the upper abdomen the day before surgical resection of the tumor. Exclusion criteria were strong artifacts that make the scan unusable for assessment of the ADC value and neoadjuvant therapy. Nineteen patients were included in this MRI cohort (= cohort 1). The demographic, clinical, and pathological characteristics of this patient cohort are summarized in Table 1.

All DW-MRI scans were acquired using a 1.5 T scanner (MAGNETOM Aera, Siemens Medical Solutions, Erlangen, Germany) with a maximum gradient strength of 45 mT/m, a six-element body-phased array coil, and a 24-channel spine array coil. Diffusion-weighted images were acquired using single-shot echo-planar imaging (SE2d-EPI) pulse sequence in expiratory breath-hold. Imaging parameters were as follows: 14 slices, slice thickness/gap = 5/0.25 mm, k-space based parallel imaging technique (GRAPPA), spectral fat saturation, acceleration factor of two. The acquisition was separated into blocks (b0, b50) and (b0, b800). Each block was acquired in a single breath-hold in expiration (TA = 22 s). 

Segmentation of the lesions on DW-MRI scans was performed independently by two board-certified radiologists, each with more than 7 years of experience in abdominal MR imaging, using the MITK Diffusion application (Medical Imaging Interaction Toolkit, Version 2017.07, DKFZ, Heidelberg, Germany; www.MITK.org). Free-hand volumes of interest (VOIs) were drawn directly on diffusion-weighted images with diffusion weighting b50 = 50 s/mm^2^ and b800 = 800 s/mm^2^. Conventional T1- and T2-weighted MR images and contrast-enhanced CT images were available in every patient and were used to improve the segmentation of the anatomical outline of the lesions. The signal magnitudes with diffusion weightings S(b50) and S(b800) were extracted, and the ADC_(50, 800)_ was calculated using the following formula, as described by Mürtz et al. [27]:

$$ADC_{\left(50,\;800\right)}=\frac{\ln\left(S\left(b50\right)\right)-\ln\left(S\left(b800\right)\right)}{b800-b50}$$

### 4.2. Patient Samples and Immunohistochemistry

Tissue samples were obtained from the tissue bank of the National Center for Tumor Diseases (NCT, Heidelberg, Germany) in accordance with the regulations of the tissue bank and the approval (primary approval: 16 Sept 2005; re-evaluation: 24 April 2015) of the ethics committee of Heidelberg University (No. 206/2005). Written informed consent of all patients was obtained. Tissue samples of 199 patients with histologically confirmed pancreatic ductal adenocarcinoma who underwent surgical resection with curative intent were analyzed on tissue microarrays, as indicated in the respective data set (cohort 2). The demographic, clinical, and pathological characteristics of this patient cohort are summarized in Table 1.

Paraffin-embedded tissue was used, and hematoxylin staining was performed. Histological analyses were performed by two board-certified surgical pathologists, with more than 10 years of diagnostic expertise in pancreas pathology. The hematoxylin-eosin (HE) stained sections were quantified for the amount of tumor and stroma by light microcopy and confirmed using an image software (Aperio ImageScope, Leica Biosystems, Nussloch, Germany). Immunohistochemical analysis was performed, using antibodies to following human antigens: IL21, IL26 (dilution: 1:100 for IL21; 1:50 for IL26; Abcam, Cambridge, UK), CD3 (dilution: 1:200; Thermo Fischer Scientific, Darmstadt, Germany,), IL10RB (dilution: 1:200; NovusBiologicals, Centenniell, Co, USA), IL20RA (dilution: 1:200; NovusBiologicals), IL21R (dilution: 1:150 NovusBiologicals), Azurocidin (dilution: 1:100; Abcam, Cambridge, UK), CD68 (ready to use antibody; DAKO, Glostrup, Denmark). Antigen retrieval was performed by heat pre-treatment using citrate buffer (pH 6.0), and antibody-binding was visualized. Histofine, simple stain universal polymer was used (Nichirei, Tokyo, Japan) followed by the color reaction with liquid permanent red (Zytomed, Berlin, Germany), or the DAKO-EnVISION Kit (DAKO) and counterstain with hematoxylin. The presence of the respective antigens was either counted (for infiltrating immune cells) or semi-quantified using the established and widely used Allred score, the sum of staining distribution (0–5 points) and intensity (0–3 points) on tumor cells, ranging from 0 (negative) to 8 (strong and ubiquitous expression) [28].

### 4.3. Pancreatic Carcinoma Cell Lines and Cell Culture

The human PDAC cell lines AsPC-1, BxPC-3, and Panc-1 were obtained from ATCC and cultivated in RPMI 1640 (Life Technologies GmbH, Darmstadt, Germany) supplemented with 10% FBS and 1% penicillin and streptomycin (*p*/S). All cells were incubated at 37 °C, with 5% CO2 and 95% humidity. For the experiments, cells were harvested when in linear growth condition. Details are described in the respective experiment.

### 4.4. Western Blotting

Cells were harvested and lysed using RIPA buffer (Santa Cruz Biotechnologies, Santa Cruz, CA, USA), and total protein content was determined using a Pierce BCA Protein Assay Kit (Thermo Fisher, Waltham, MA USA). Twenty micrograms total protein was loaded on SDS-PAGE (10%); a prestained Rec Protein Ladder (Thermo Fisher) was used as a molecular weight marker. For Western blotting, proteins were transferred to a 0.45 μm nitrocellulose membrane (GE Healthcare GmbH, Freiburg, Germany) and 5% skim milk (Carl Roth GmbH, Karlsruhe, Germany) or 3% BSA (Sigma–Aldrich GmbH, Taufkirchen, Germany) were used for blocking. The following primary anti-human antibodies were used: rabbit anti-human IL10RB (dilution: 1:400; NovusBiologicals), mouse anti-human IL20RA (dilution: 1:400; Novus Biologicals), rabbit anti-human ERK (dilution: 1:1000; Cell Signaling Technology), rabbit anti-human pERK (dilution: 1:1000; Cell Signaling Technology), rabbit anti-human STAT3 (dilution: 1:1000; Cell Signaling Technology), rabbit anti-human pSTAT3 (dilution: 1:1000; Cell Signaling Technology), mouse anti-human *β*-actin (dilution: 1:10000; MP Biomedicals, Eschwege, Germany) and mouse anti-human *β*-tubulin (dilution: 1:300; Santa Cruz Biotechnologies). Following incubation at 4 °C overnight, antibody binding was assessed using fluorescent donkey anti-rabbit or donkey anti-mouse secondary antibodies, all in a dilution of 1:20000 (LI-COR^®^ Biosciences GmbH, Bad Homburg, Germany). Protein bands were visualized using a LI-COR^®^ Imaging Systems (LI-COR^®^ Biosciences GmbH) and documented by LI-COR^®^ Image Studio™ Software. For each experimental condition, at least three independent experiments were performed.

### 4.5. Immunofluorescence Staining of IL10RB, IL20RA, and IL21R

Pancreatic cancer cells (50 × 10^3^ / 500 µL) were seeded into Lab-Tek^®^ Chamber Slides (Thermo Fisher). After 24 h, cells were fixed with 2 % PFA and incubated with a rabbit anti-human IL10RB antibody (dilution: 1:50; NBP2–33492, NovusBiologicals) and a mouse anti-human IL20RA antibody (dilution: 1:50; NBP2–11695, Novus Biologicals), at 4 °C overnight. As secondary antibodies, an Alexa488-conjugated goat anti-mouse antibody (dilution: 1:200; Dianova, Jackson, Hamburg, Germany) or an Alexa568-conjugated donkey anti-rabbit antibody (dilution:1:500; Thermo Fisher Scientific) was used. Slides were mounted with a coverslip using ProLong^®^ Diamond Antifade Mountant with DAPI (Thermo Fisher) and viewed by digital microscopy (Carl Zeiss Microscopy, Jena, Germany) and pictures captured by a camera (Leica, Wetzlar, Germany).

### 4.6. Colony Formation Assay

1 × 10^4^ cells of AsPC-1, 1.7 × 10^4^ cells of BxPC-3, and 0.3 × 10^4^ cells of Panc-1 were seeded in a 6-well plate. The following day, cells were treated with IL21 or IL26, respectively (10 ng/mL). Plates were incubated for 12 days; cytokine medium was changed twice. The medium was aspirated, and wells were washed with PBS. One milliliter ice-cold methanol was added to each well, and cells were fixed for 10 min at −20 °C. One milliliter crystal violet solution (0.05 % *w*/*v*) was added to each well and incubated at a plate reader for 10 min at RT. Crystal violet was aspirated, plates were washed with purified water, and dried covered overnight. Colony formation was documented using AlphaImager MultiImage Light Cabinet (Alpha-InnoTec GmbH, Kasendorf, Germany) and analyzed with ImageJ. For each experimental condition, at least three independent experiments were performed.

### 4.7. Statistical Analysis

Statistical data analysis was performed using MedCalc Version 19.1 (MedCalc Software, Ostend, Belgium). Inter-reader reliability of DW-MRI analysis was assessed by using the intra-class correlation coefficient (ICC) and applying a 2-way ICC with random raters’ assumption reproducibility. The Mann–Whitney U test for independent samples was used to compare a) the numbers of IL21^+^ or IL26^+^ cells/mm^2^ between tumors with low ADC_(50, 800)_ values (<1.3 × 10^–3^ mm^2^/sec) versus tumors with high ADC_(50, 800)_ values (≥1.3 × 10^−3^ mm^2^/sec), b) the ADC_(50, 800)_ values between patients with high versus low numbers of IL21^+^ or IL26^+^ cells/mm^2^, and c) the numbers of CD68^+^ cells or Azurocidin^+^ cells between patients with high IL10RB AND IL20RA Allred scores (≥5) versus all other patients. The numbers of IL21^+^/ IL26^+^ cells/mm^2^ were compared between patients with a) small versus large tumors (T1/2 versus T3), b) with versus without lymph node metastases (N+ versus N0), and c) low/ intermediate versus high grading (G1/2 versus G3).

The colony numbers of AsPC-1, BxPC-3, and Panc-1 cells were compared between untreated controls and cells treated with IL21 or IL26, after normalization to the mean colony number of the untreated controls on the respective day, using the Mann–Whitney U test. 

Spearman rank correlation coefficients were calculated between the numbers of IL21^+^/IL26^+^ cells and the number of CD3^+^ cells. Moreover, rank correlation was performed to investigate the relationship between IL10RB and IL20RA expression (Allred score, distribution, and intensity). 

Overall survival analyses were performed. Fifteen patients who died within the first 60 days after surgery were excluded from the survival analyses because complications from surgery were considered the likeliest cause of death. One hundred and eighty-four patients were included in survival analysis. Median follow-up time was 577 days (range: 75 to 1801 days). Kaplan–Meier estimates were calculated for overall survival analysis of patients with low versus high number of CD3^+^ cells (<20/mm^2^ versus ≥20/mm^2^), low versus high number of IL21^+^ cells or IL26^+^ cells (<10/mm^2^ versus ≥ 10/mm^2^), low versus high IL10RB, IL20RA, and/or IL21R Allred scores (0–4 versus 5–8). The log-rank test was used to analyze the statistical significance of Kaplan–Meier estimates. 

*p* < 0.05 were considered to be statistically significant.

## Figures and Tables

**Table 1:** Demographic, clinical, and pathological characteristics of all patients.

	Cohort 1 (*n* = 19)	Cohort 2 (*n* = 199)
Sex		
Female	12	98
Male	7	101
Mean Age ± SD [years]	66.2 ± 8.1	64.1 ± 9.8
Location of primary tumor		
Pancreatic head	14	192
Pancreatic body and/ or tail	5	7
Type of surgery		
Pylorus-preserving Whipple	6	167
Classical Whipple	4	21
Left pancreatic resection	3	5
Total pancreatectomy	6	6
Extent of primary tumor (T)		
T1	0	23
T2	0	155
T3	19	21
T4	0	0
Lymph node metastases (N)		
N0	5	32
N1	4	70
N2	10	97
Distant metastases (M)		
M0/Mx	19	190
M1	0	9
Tumor Grading (G)		
G1	0	5
G2	11	126
G3	8	68

## Supplementary Materials

The following are available online at https://www.mdpi.com/1422-0067/21/1/346/s1.

## Abbreviations

ADC	Apparent diffusion coefficient
BCA	Bicinchoninic acid
BSA	Bovine serum albumin
CD	Cluster of differentiation
CT	Computed tomography
DAPI	4′,6-diamidino-2-phenylindole
DW	Diffusion-weighted
DWI	Diffusion-weighted imaging
ERK	Extracellular signal-related kinase
FOLFIRINOX	Combination therapy containing folinic acid, fluorouracil, irinotecan, and oxaliplatin
GRAPPA	Generalized autocalibrating partial parallel acquisition
HASTE	Half-Fourier acquisition single-shot turbo spin echo
HE	Hematoxylin-eosin
ICC	Intraclass correlation coefficient
IHC	Immunohistochemistry
IL	Interleukin
JAK	Janus Kinase
MITK	Medical Imaging Interaction Toolkit
MRI	Magnetic resonance imaging
NCT	National Center for Tumor Diseases
OS	Overall survival
PBS	Phosphate-buffered saline
PDAC	Pancreatic ductal adenocarcinoma
PFA	Paraformaldehyde
RIPA	Radioimmunoprecipitation assay
RIS	Radiological information system
r_s_	Spearman rank correlation coefficient
RT	Room temperature
S	Signal magnitude
SDS-PAGE	Sodium dodecyl sulfate-polyacrylamide gel electrophoresis
SE2d-EPI	Single-shot two-dimensional echo-planar imaging
STAT	Signal transducer and activator of transcription
T	Tesla
TNF	Tumor necrosis factor
VOI	Volume of interest

## References

[1] Siegel R.L., Miller K.D., Jemal A. (2019). Cancer statistics, 2019. CA. Cancer J. Clin..

[2] Coussens L.M., Zitvogel L., Palucka A.K. (2013). Neutralizing tumor-promoting chronic inflammation: a magic bullet?. Science.

[3] Kleeff J., Beckhove P., Esposito I., Herzig S., Huber P.E., Löhr J.M., Friess H. (2007). Pancreatic cancer microenvironment. Int. J. Cancer.

[4] Wente M.N., Gaida M.M., Mayer C., Michalski C.W., Haag N., Giese T., Felix K., Bergmann F., Giese N.A., Friess H. (2008). Expression and potential function of the CXC chemokine CXCL16 in pancreatic ductal adenocarcinoma. Int. J. Oncol..

[5] Swayden M., Iovanna J., Soubeyran P. (2018). Pancreatic cancer chemo-resistance is driven by tumor phenotype rather than tumor genotype. Heliyon.

[6] Sapalidis K., Kosmidis C., Funtanidou V., Katsaounis A., Barmpas A., Koimtzis G., Mantalobas S., Alexandrou V., Aidoni Z., Koulouris C. (2019). Update on current pancreatic treatments: from molecular pathways to treatment. J. Cancer.

[7] Falzone L., Salomone S., Libra M. (2018). Evolution of Cancer Pharmacological Treatments at the Turn of the Third Millennium. Front. Pharmacol..

[8] Alam M.S., Gaida M.M., Bergmann F., Lasitschka F., Giese T., Giese N.A., Hackert T., Hinz U., Hussain S.P., Kozlov S.V. (2015). Selective inhibition of the p38 alternative activation pathway in infiltrating T cells inhibits pancreatic cancer progression. Nat. Med..

[9] McAllister F., Bailey J.M., Alsina J., Nirschl C.J., Sharma R., Fan H., Rattigan Y., Roeser J.C., Lankapalli R.H., Zhang H. (2014). Oncogenic Kras activates a hematopoietic-to-epithelial IL-17 signaling axis in preinvasive pancreatic neoplasia. Cancer Cell.

[10] Linnebacher A., Mayer P., Marnet N., Bergmann F., Herpel E., Revia S., Yin L., Liu L., Hackert T., Giese T. (2019). Interleukin 21 Receptor/Ligand Interaction Is Linked to Disease Progression in Pancreatic Cancer. Cells.

[11] He W., Wu J., Shi J., Huo Y.-M., Dai W., Geng J., Lu P., Yang M.-W., Fang Y., Wang W. (2018). IL22RA1/STAT3 Signaling Promotes Stemness and Tumorigenicity in Pancreatic Cancer. Cancer Res..

[12] Rao C.V., Mohammed A., Janakiram N.B., Li Q., Ritchie R.L., Lightfoot S., Vibhudutta A., Steele V.E. (2012). Inhibition of pancreatic intraepithelial neoplasia progression to carcinoma by nitric oxide-releasing aspirin in p48(Cre/+)-LSL-Kras(G12D/+) mice. Neoplasia N. Y..

[13] Mayorek N., Naftali-Shani N., Grunewald M. (2010). Diclofenac inhibits tumor growth in a murine model of pancreatic cancer by modulation of VEGF levels and arginase activity. PLoS ONE.

[14] Türkbey B., Aras Ö., Karabulut N., Turgut A.T., Akpinar E., Alibek S., Pang Y., Ertürk Ş.M., El Khouli R.H., Bluemke D.A. (2012). Diffusion-weighted MRI for detecting and monitoring cancer: a review of current applications in body imaging. Diagn. Interv. Radiol. Ank. Turk..

[15] Fukukura Y., Takumi K., Kamimura K., Shindo T., Kumagae Y., Tateyama A., Nakajo M. (2012). Pancreatic adenocarcinoma: variability of diffusion-weighted MR imaging findings. Radiology.

[16] Klauss M., Gaida M.M., Lemke A., Grünberg K., Simon D., Wente M.N., Delorme S., Kauczor H.-U., Grenacher L., Stieltjes B. (2013). Fibrosis and pancreatic lesions: counterintuitive behavior of the diffusion imaging-derived structural diffusion coefficient d. Investig. Radiol..

[17] Muraoka N., Uematsu H., Kimura H., Imamura Y., Fujiwara Y., Murakami M., Yamaguchi A., Itoh H. (2008). Apparent diffusion coefficient in pancreatic cancer: characterization and histopathological correlations. J. Magn. Reson. Imaging JMRI.

[18] Heid I., Steiger K., Trajkovic-Arsic M., Settles M., Eßwein M.R., Erkan M., Kleeff J., Jäger C., Friess H., Haller B. (2017). Co-clinical Assessment of Tumor Cellularity in Pancreatic Cancer. Clin. Cancer Res. Off. J. Am. Assoc. Cancer Res..

[19] Barral M., Taouli B., Guiu B., Koh D.-M., Luciani A., Manfredi R., Vilgrain V., Hoeffel C., Kanematsu M., Soyer P. (2015). Diffusion-weighted MR imaging of the pancreas: current status and recommendations. Radiology.

[20] Mayer P., Dinkic C., Jesenofsky R., Klauss M., Schirmacher P., Dapunt U., Hackert T., Uhle F., Hänsch G.M., Gaida M.M. (2018). Changes in the microarchitecture of the pancreatic cancer stroma are linked to neutrophil-dependent reprogramming of stellate cells and reflected by diffusion-weighted magnetic resonance imaging. Theranostics.

[21] Wiggermann P., Grützmann R., Weissenböck A., Kamusella P., Dittert D.-D., Stroszczynski C. (2012). Apparent diffusion coefficient measurements of the pancreas, pancreas carcinoma, and mass-forming focal pancreatitis. Acta Radiol. Stockh. Swed. 1987.

[22] Lemke A., Laun F.B., Klauss M., Re T.J., Simon D., Delorme S., Schad L.R., Stieltjes B. (2009). Differentiation of pancreas carcinoma from healthy pancreatic tissue using multiple b-values: comparison of apparent diffusion coefficient and intravoxel incoherent motion derived parameters. Invest. Radiol..

[23] Barilla R.M., Diskin B., Caso R.C., Lee K.B., Mohan N., Buttar C., Adam S., Sekendiz Z., Wang J., Salas R.D. (2019). Specialized dendritic cells induce tumor-promoting IL-10+IL-17+ FoxP3neg regulatory CD4+ T cells in pancreatic carcinoma. Nat. Commun..

[24] Tengvall S., Che K.F., Lindén A. (2016). Interleukin-26: An Emerging Player in Host Defense and Inflammation. J. Innate Immun..

[25] Hör S., Pirzer H., Dumoutier L., Bauer F., Wittmann S., Sticht H., Renauld J.-C., de Waal Malefyt R., Fickenscher H. (2004). The T-cell lymphokine interleukin-26 targets epithelial cells through the interleukin-20 receptor 1 and interleukin-10 receptor 2 chains. J. Biol. Chem..

[26] You W., Tang Q., Zhang C., Wu J., Gu C., Wu Z., Li X. (2013). IL-26 promotes the proliferation and survival of human gastric cancer cells by regulating the balance of STAT1 and STAT3 activation. PLoS ONE.

[27] Mürtz P., Sprinkart A.M., Reick M., Pieper C.C., Schievelkamp A.-H., König R., Schild H.H., Willinek W.A., Kukuk G.M. (2018). Accurate IVIM model-based liver lesion characterisation can be achieved with only three b-value DWI. Eur. Radiol..

[28] Allred D.C., Clark G.M., Elledge R., Fuqua S.A., Brown R.W., Chamness G.C., Osborne C.K., McGuire W.L. (1993). Association of p53 protein expression with tumor cell proliferation rate and clinical outcome in node-negative breast cancer. J. Natl. Cancer Inst..

