# Supplementary material for: The Microarchitecture of Pancreatic Cancer as Measured by Diffusion-Weighted Magnetic Resonance Imaging Is Altered by T Cells with a Tumor Promoting Th17 Phenotype
Source: Int J Mol Sci. 2020 Jan 5;21(1):346. doi: 10.3390/ijms21010346 (PMC6982276; doi:10.3390/ijms21010346)
Supplement: Supplementary file 1 [file ijms-21-00346-s001.pdf]

Supplementary Materials:

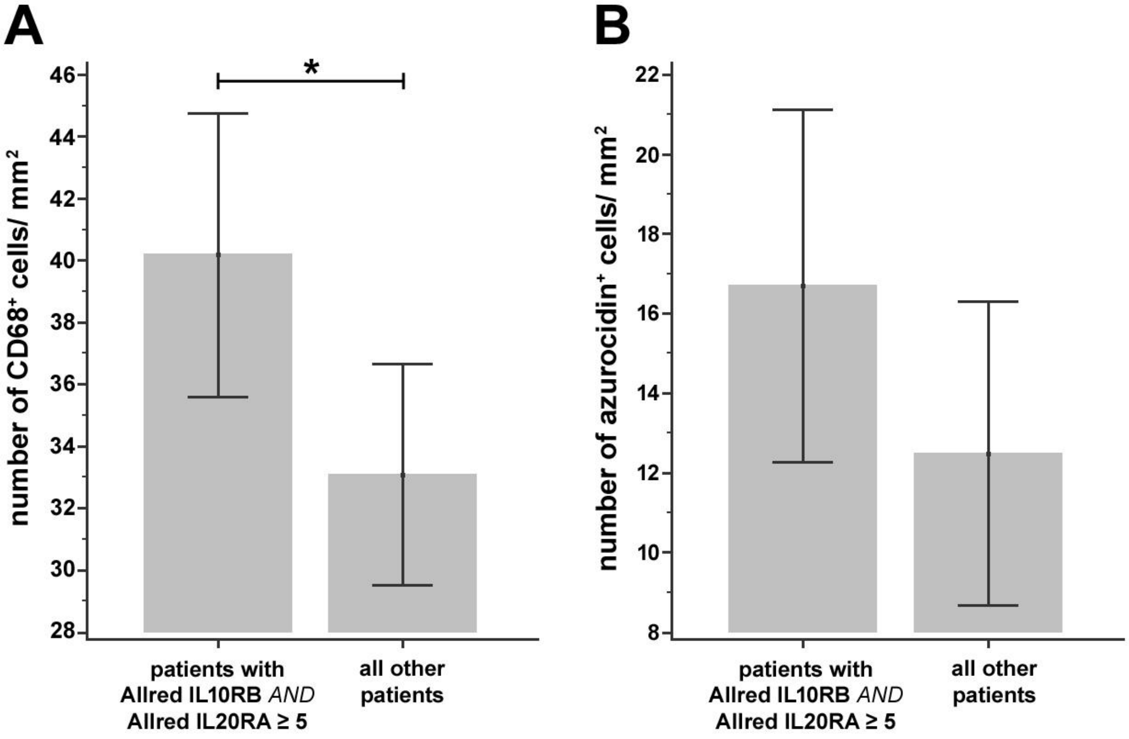

Supplementary Figure 1. Inflammation depending on expression of IL10RB and IL20RA. A) Number of CD68+ cells/mm2 was higher in patients with Allred IL10RB AND Allred IL20RA compared to all other patients. B) Azurocidin+ infiltrate was higher in patients with Allred IL10RB AND Allred IL20RA compared to all other patients. Differences that are statistically different ($p < 0.05$) according to the Mann-Whitney U test are marked with *.
